# Sensitivity, specificity, and reproducibility of RNA-Seq differential expression calls

**DOI:** 10.1186/s13062-016-0169-7

**Published:** 2016-12-20

**Authors:** Paweł P. Łabaj, David P. Kreil

**Affiliations:** 1 APART Fellow, Austrian Academy of Science, Vienna, Austria; 2 Chair of Bioinformatics Research Group, Boku University, Vienna, Austria

**Keywords:** RNA-seq, Sensitivity, Specificity, Reproducibility, Differential expression calling

## Abstract

**Background:**

The MAQC/SEQC consortium has recently compiled a key benchmark that can serve for testing the latest developments in analysis tools for microarray and RNA-seq expression profiling. Such objective benchmarks are required for basic and applied research, and can be critical for clinical and regulatory outcomes. Going beyond the first comparisons presented in the original SEQC study, we here present extended benchmarks including effect strengths typical of common experiments.

**Results:**

With artefacts removed by factor analysis and additional filters, for genome scale surveys, the reproducibility of differential expression calls typically exceed 80% for all tool combinations examined. This directly reflects the robustness of results and reproducibility across different studies. Similar improvements are observed for the top ranked candidates with the strongest relative expression change, although here some tools clearly perform better than others, with typical reproducibility ranging from 60 to 93%.

**Conclusions:**

In our benchmark of alternative tools for RNA-seq data analysis we demonstrated the benefits that can be gained by analysing results in the context of other experiments employing a reference standard sample. This allowed the computational identification and removal of hidden confounders, for instance, by factor analysis. In itself, this already substantially improved the empirical False Discovery Rate (eFDR) without changing the overall landscape of sensitivity. Further filtering of false positives, however, is required to obtain acceptable eFDR levels. Appropriate filters noticeably improved agreement of differentially expressed genes both across sites and between alternative differential expression analysis pipelines.

**Reviewers:**

An extended abstract of this research paper was selected for the Camda Satellite Meeting to Ismb 2015 by the Camda Programme Committee. The full research paper then underwent one round of Open Peer Review under a responsible Camda Programme Committee member, Lan Hu, PhD (Bio-Rad Laboratories, Digital Biology Center-Cambridge). Open Peer Review was provided by Charlotte Soneson, PhD (University of Zürich) and Michał Okoniewski, PhD (ETH Zürich). The Reviewer Comments section shows the full reviews and author responses.

**Electronic supplementary material:**

The online version of this article (doi:10.1186/s13062-016-0169-7) contains supplementary material, which is available to authorized users.

## Background

The MAQC [1] and SEQC [2, 3] consortia have over the years compiled key resources for testing the performance of experimental platforms and computational analysis tools for expression profiling. Such objective benchmarks are required for effective research as well as clinical and regulatory applications. In this study, based on the latest SEQC data sets, we investigate the sensitivity, specificity, and reproducibility of RNA-seq differential expression calls. Going beyond the first comparisons presented in the original SEQC study [2, 3], we here present extended benchmarks including effect strengths typical of common experiments. In particular we focus on comparisons of SEQC standardized reference samples A and C, where C consists of 3 parts of sample A and 1 part of sample B (see Methods) [1, 2]. This pair of samples has the smallest average effect strength (signal) amongst the different possible pair-wise comparisons of the MAQC/SEQC samples A, B, C, and D, allowing us to also consider performance for more subtle signals, such as expression changes for typically weakly expressed molecular switches. For a comprehensive benchmark of alternative methods for differential expression analysis, we here consider all 55,674 known human genes [4], for an unbiased assessment of the impact of RNA-seq pipeline choice. Our comparison of selected tools represents the wide range of algorithms currently available for gene expression estimation and differential expression calling, reflecting the rapid development of the field. The studied metrics cover sensitivity, specificity, and reproducibility. Our benchmark tests both the consistency of results from genome wide screens or surveys as well as the robust identification of the top ranked candidates with the strongest relative expression change.

## Methods

### Experimental study design and data

This study builds on the main synthetic benchmark data set of the SEQC consortium, where known mixtures of standardized reference samples have been sequenced by multiple platforms in a setup controlling for laboratory site specific effects [2]. In particular, the well-characterized reference RNA samples A (Universal Human Reference RNA) and B (Human Brain Reference RNA) from the MAQC consortium have been used [1]. Samples A and B were then mixed in known ratios, 3:1 and 1:3, to construct samples C and D, respectively. In this data analysis benchmark our results are based on the subset of samples A and C at six Illumina HiSeq 2000 sites where each sample has been sequences with 4 technical replicates.

### Gene expression profiling

In this study the AceView gene models have been used [4]. We previously have shown that, despite its age, AceView remains the most comprehensive and accurate annotation database for human [2]. The expression profiles of human AceView genes have been assessed by selected tools representing the state of the art in expression profiling analysis. Expression estimates are represented in the form of read count equivalents. r-make (based on STAR) [5] and Subread [6] performs an alignment of sequenced reads to the genome, followed by counting reads that fall into known gene regions. The popular TopHat2 tool [7] with the ‘–G’ option pursues a hybrid approach, where based on the provided gene model the virtual transcriptome is constructed and reads are first aligned to it, in line with our earlier analysis first showing that this improves the precision of expression estimates [8]. In the next steps these aligned reads are mapped back to the genome and the remaining not aligned yet reads are aligned to the genome sequences. Gene and transcript expression levels are then estimated using the matching Cufflinks2 [9] tool that processes the genome-based alignments. In contrast, BitSeq [10] directly uses the transcriptome alignments (here we have aligned the reads to the transcriptome with use of SHRiMP2 [11]) to assess transcript abundances. These are then summarized to obtain expression level estimates for genes. kallisto [12] takes an alignment free approach, where transcript abundances are estimated directly from reads based on the idea of pseudo-alignment for rapidly determining the compatibility of reads with target transcript sequences, without the need for a full alignment. This lightweight approach has been motivated by Sailfish [13] which is not considered here. Transcript expression estimates are again summarized to obtain expression estimates for genes. This approach of obtaining gene level estimates from transcript level results has recently been found to improve gene-level inference in differential analyses for RNA-seq [14]. Details of how all tools were run can be found in the Supplementary materials of the original SEQC/MAQC-III study; [2] kallisto has been used with default parameters.

### Factor analysis

Factor analysis was performed to remove unwanted variation. We examined the tool svaseq [15], which provides SVA [16] with adaptations for RNA-seq data. SVA [16] together with PEER [17]were the leading preprocessing tools of the original SEQC study [3]. Gene expression estimates for all samples were used to detect latent variables. Co-variates associated with sample type were included for inference and the inferred hidden confounders were removed from the signal.

### Differential expression calls

In differential expression analysis of samples A/C we can focus on genes down-regulated in sample A because the effect strength of any potential up-regulation is limited to maximum of 4/3-fold increase by design, as sample C is 3 parts of sample A and one part of sample B. We therefore expect no up-regulated genes satisfying commonly used thresholds for effect strength.

We examined the effect of method choice in differential expression analysis by comparison of three popular alternatives: limma [18], edgeR [19, 20], and DESeq2 [21], each of which has been run with default settings. For instance, limma by default includes TMM[20]-normalization and voom[22] preprocessing. The FDR was controlled by Benjamini-Hochberg adjustment for multiple testing. Genes were called differentially expressed for *q*<5*%*. Additional filter rules were optionally applied, requiring a minimum effect strength of 1 (i.e., | log2(*F* *C*)|>1, meaning a fold change larger than 2). In addition, the optional filter required an Average Expression above a specific threshold. This threshold was defined for each combination of methods for expression estimation and differential expression calling so as to equalize intra-site sensitivity after svaseq correction. It was set so that for an average site 3,000 genes were identified as differentially expressed. The same thresholds have been applied to inter-site differential expression calls. Once the effect strength filter has been applied, even dropping the 45% least strongly expressed genes removes only 16% of the remaining differential expression calls (Tables 1 and 2), which constitutes just 2.5% of all AceView genes.

**Table 1:** Differential expression calls

EE	DEC	raw	sva	sva+FC	sva+FC+AE
r-Make	limma	7226	8078	4498 [56%]	3058 [38%]
	edgeR	7314	8720	4908 [56%]	3058 [35%]
	DESeq2	6974	8380	4552 [54%]	3060 [37%]
Subread	limma	9772	9557	4795 [50%]	3016 [32%]
	edgeR	10202	10522	5398 [51%]	3036 [29%]
	DESeq2	9308	9709	4662 [48%]	3052 [31%]
TopHat2/	limma	8854	8782	4450 [51%]	3058 [35%]
Cufflinks2	edgeR	7329	7104	4386 [62%]	3018 [42%]
	DESeq2	8536	8489	4077 [48%]	3061 [36%]
SHRiMP2/	limma	8952	8276	4086 [49%]	3045 [37%]
BitSeq	edgeR	8791	8663	4526 [52%]	3025 [35%]
	DESeq2	7590	7878	3804 [48%]	3038 [39%]
kallisto	limma	8984	8851	4410 [50%]	3022 [34%]
	edgeR	9356	9284	4666 [50%]	3039 [33%]
	DESeq2	8016	8296	3915 [47%]	3044 [37%]

The table displays the number of differential expression calls, reflecting sensitivity, as obtained after specific analysis steps. For all combinations of methods for expression estimation and differential expression calling, we compare the typical numbers of genes classified as differentially expressed (*q*<5*%*). The columns show median results across sites for: raw expression estimates; expression estimates after svaseq correction; expression estimates after svaseq correction and application of additional filters for effect strength, i.e., fold-change (|log2*F* *C*|>1); and expression estimates after svaseq correction and application of additional filters for effect strength (|log2*F* *C*|>1) and minimum average expression (AE thresholds in Table 2). The last two columns also give a percentage relative to the numbers of genes found after svaseq correction and no additional filters. This highlights that the additional filtering for weak expression removes only a further 16% of genes originally classified as differentially expressed in addition to the ones already removed by the usual filters for log-fold change, affecting just 2.5% of all genes

**Table 2:** Average expression thresholds

EE\DEC	limma	edgeR	DESeq2
Subread	42.6	46.6	45.4
r-Make	42.6	48.1	46.8
TopHat2/Cufflinks2	42.6	47.8	46.1
SHRiMP2/BitSeq	41.6	47.8	45.1
kallisto	41.3	46.4	42.8

Our benchmark compares the specificity and reproducibility of differential expression analysis for different tools. For a meaningful comparison, all tools are run to give the same sensitivity. For each combination of methods for expression estimation (EE) and differential expression calling (DEC), a threshold for removing the most weakly expressed genes was therefore determined to adjust sensitivity as required. The percentile of genes filtered is shown for which 3,000 genes were found at an average site (*q*<5*%* and absolute log-fold change larger than one)

### Empirical false discovery rate

Taking advantage of the SEQC study design [2]we can infer an empirical False Discovery Rate (eFDR) by comparing the amount of genes identified as differentially expressed in the cross-site same–same comparison (A-*vs*-A and C-*vs*-C) with the differentially expressed genes in the A-*vs*-C comparison: *e* *F* *D* *R*=(*A*_1_−*v* *s*−*A*_2_+*C*_1_−*v* *s*−*C*_2_)/(*A*_1_−*v* *s*−*C*_2_+*A*_2_−*v* *s*−*C*_1_), where: *X*_*N*_−*v* *s*−*Y*_*M*_ is the number of genes identified as differentially expressed when comparing sample *X* from site *N* with sample *Y* from site *M*.

### Inter-site reproducibility

The overall agreement between lists of differentially expressed genes has been calculated as the ratio of list intersection and list union. The agreement of the top *N* candidates has been calculated as the ratio of the length of the intersection of the top *N* genes from the compared lists (differentially expressed candidates have been order by effect strength) divided by *N*. The direction of fold change is taken into account: genes showing opposite directions of change are considered not to agree, and are thus excluded for computing the list intersection assessing agreement. All gene lists are sets, either including or excluding gene names, with no gene counted more than once.

## Results

In our benchmark analysis we investigated a wide range of tools for differential expression analysis. This analysis typically includes two steps, that are sometimes performed by different tools: estimation of gene/transcript expression levels, and significance calls for differential expression. Our comparative benchmark assessed a representative selection of tools for expression estimation, including r-make [5], Subread [6], TopHat2/Cufflinks2 [7, 9], SHRiMP2/BitSeq [10, 11], and kallisto [12], in combination with several established tools for differential expression calling, including limma [18, 22, 23], edgeR [19, 20], and DESeq2 [21]. While new tools are rapidly emerging in the field, the selection in our comparative survey covers the main approaches in the current state of the art of RNA-seq analysis for differential expression analysis.

### Differential expression call sensitivity

**Fig. 1:**
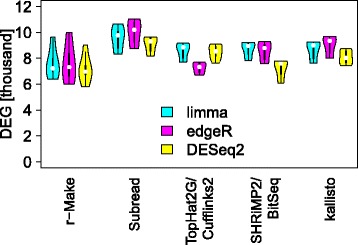
Intra-site differential expression calls. At each site, we identify genes differentially expressed between samples A and C. The *y*-axis [DEG] shows the number of significant differential expression calls (*q*<5*%*), reflecting sensitivity. Violin plots summarize the results for all sites. Plots for various methods of expression estimation are shown along the *x*-axis, with methods for differential expression calling indicated by colour

**Fig. 2:**
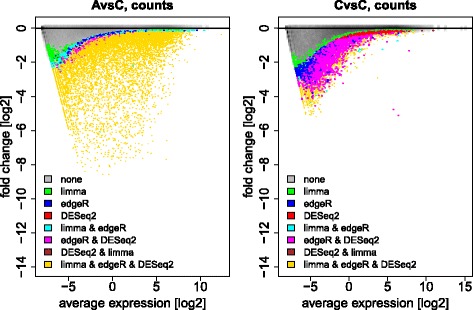
Traditional *M*(*A*) plots for A-*vs*-C and A-*vs*-A comparisons. The left panel displays the overlap of differential expression calls by different methods for an A-*vs*-C comparison, while the right panel shows results for a C-*vs*-C comparison. Partial agreement between different methods is indicated by different colours (see legend). Grey clouds represent unregulated genes. Plots show data for a typical site for read counts after normalization but without correction by factor analysis or any filters

Depending on the methods employed, the numbers of genes called differentially expressed vary roughly between 6 and 11 thousand (Fig. 1 and Table 1). To investigate this further we examined *M*(*A*) plots, where genes are represented by dots coloured according to which methods identified them as differentially expressed. Figure 2 shows typical *M*(*A*) plots observed. In an A-*vs*-C comparison (left panel) we can identify areas where different methods are particularly sensitive. Variations in the sensitivity of methods for different effect strengths (*M*) and gene abundances (*A*) reflect the range of alternative approaches to data normalization and statistics used for differential expression calling. Among the examined methods, DESeq2 seems to be the most conservative in calling genes of low average expression differentially expressed. This may be appropriate considering the relatively high variance of low count data that is characteristic of weakly expressed genes in RNA-seq [8]. Moreover, a same–same comparison (C-*vs*-C, Fig. 2 right panel) demonstrates that weakly expressed genes are also more strongly affected by random site-to-site variation, which we have shown to be largely due to the library preparation step [3]. Genes identified as differentially expressed in this same–same comparison constitute false positives in a search for biologically relevant differences, allowing us to infer an empirical False Discovery Rate (eFDR) from this benchmark to also assess specificity.

### Specificity improvements

**Fig. 3:**
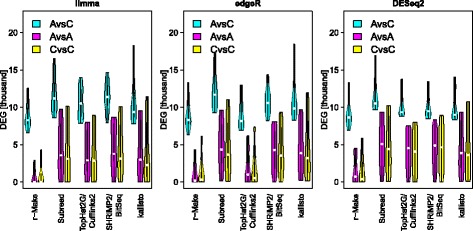
Inter-site differential expression calls. We identify genes differentially expressed between samples from alternative sites. The *y*-axis [DEG] shows the number of differential expression calls (*q*<5*%*). Violin plots summarize the results for all possible pairs of alternative sites. Each panel shows data for a particular method of differential expression calling. Plots for various methods of expression estimation are shown along the *x*-axis. Colour indicates the samples compared: A-*vs*-C (*cyan*), A-*vs*-A (*magenta*), and C-*vs*-C. High counts in same–same comparisons reflect a lack of specificity

**Fig. 4:**
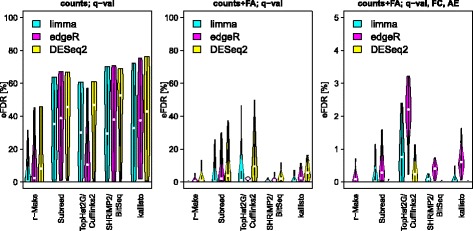
Empirical False Discovery Rate (eFDR). We estimate an eFDR by dividing the number of differential expression calls in inter-site A-*vs*-A and C-*vs*-C comparisons by the number of calls in A-*vs*-C comparisons. The left panel shows the original results for *q*-value thresholding only (no additional processing or filters). In the middle panel, hidden confounders have been removed by svaseq. In the right panel, additional filters have also been applied. Plots for various methods of expression estimation are shown along the *x*-axis, with methods for differential expression calling indicated by colour

**Fig. 5:**
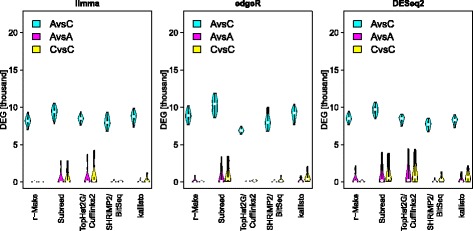
Inter-site differential expression calls after removing unwanted variation with svaseq. We identify genes differentially expressed between samples from alternative sites. The *y*-axis [DEG] shows the number of differential expression calls (*q*<5*%*). Violin plots summarize the results for all possible pairs of alternative sites. Each panel shows data for a particular method of differential expression calling. Plots for various methods of expression estimation are shown along the *x*-axis. Colour indicates the samples compared: A-*vs*-C (*cyan*), A-*vs*-A (*magenta*), and C-*vs*-C. High counts in same–same comparisons reflect a lack of specificity. Note the improvements relative to Fig. 3

We can calculate an empirical False Discovery Rate (eFDR) by comparing the cross-site sensitivities for A-*vs*-C, C-*vs*-C, and A-*vs*-A comparisons (Fig. 3 and Fig. 4 left panel). Notably, over two thousand false positives were identified in cross-site same–same comparisons (A-*vs*-A or C-*vs*-C), irrespective of the employed computational analysis tools. In some cases over ten thousand false positives were found, approaching the number of differential expression calls in A-*vs*-C comparisons. Consequently, without further processing, high eFDRs are observed. The number of false positives can be reduced when unwanted variation is removed [16, 17]. For this, experimental results must be analysed in the context of similar experiments, e. g., from public repositories. In our study we can use different sequencing sites to provide such a context. Applying svaseq[15] to remove unwanted variation we could achieve a drastic reduction of false positives (Fig. 5). This was achieved without a change to the overall sensitivity landscape of the A-*vs*-C comparison (see Table 1 for intra-site and Fig. 5 *vs* Fig. 3 for inter-site A-*vs*-C comparisons). As a result the eFDR could be improved from 30−50*%* to typically below 10% (Fig. 4 left *vs* middle panel). Even after svaseq, however, we observed some instances of eFDRs up to 50% (outlier sites for TopHat2/Cufflinks2). For reliable differential expression analysis, a further improvement of eFDR levels is thus needed. Additional filtering steps have been successfully used to that effect [1–3, 24]. For RNA-seq, unlike for microarrays, beyond filters for small effect size (fold change) also filters for small expression levels are necessary. This is needed in order to remove False Positives arising from the large scatter for weakly expressed transcripts, which can be seen as a ’comet head’ in typical *M*(*A*) plots (Fig. 2). With appropriate additional filters, the eFDR could consistently be reduced below 5%. Except for the combination of TopHat2/Cufflinks2 with edgeR, the typical eFDR even dropped below 1% (Fig. 4).

### Effects on implicated genes

**Fig. 6:**
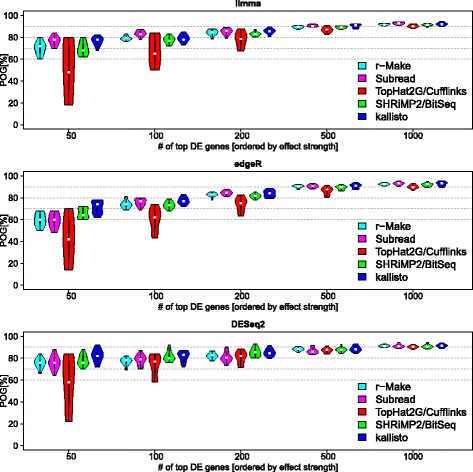
Inter-site reproducibility of differential expression calls. We assess the reproducibility of the top ranked differentially expressed genes across sites. The *y*-axis plots the percentage of genes (POG) identified as differentially expressed in the same direction and with significance in both alternative sites compared. We investigate this for the 50 top-ranked genes on the left of the plot, and consider larger lists going to the right along the *x*-axis. The violin plots summarize the results for all possible pairs of alternative sites. The observed pipeline specific effects were more pronounced for the shorter lists, which typically are of more immediate relevance in a search for leads or biomarkers. Agreement for the top 1000 genes was above 90% irrespective of pipeline choice. Results for BitSeq or kallisto and DESeq2 were also robust for shorter lists. Hidden confounders were removed from expression estimates by svaseq, and additional filters for average expression and effect strength were applied for differential expression calls. Genes meeting criteria for differential expression calls were ranked by effect size (| log2*F* *C*|)

The goal of many studies in the medical and the life sciences is to identify pathways of interest by differential expression profiling. Comprehensive lists of differentially expressed genes which can reliably be reproduced by other laboratories are central to this widely employed approach. In site-to-site comparisons of the genes for which significant differential expression was identified, agreement ranged from 70−76*%*, depending on the employed methods. Application of additional filters for effect size and abundance improved agreement to 79−85*%*. Similarly, in method-to-method comparisons, agreement was typically not higher than 64% even after application of svaseq (cf. Fig. 2 a). The additional filters improved this to 86−91*%*. Notably, however, research interest is often focused on the genes with the strongest fold change. Using so-called ‘violin plots’ to represent the distributions of results, Fig. 6 plots the percentage agreement across sites (*y*-axis) for the *N* top ranked differentially expressed genes sorted by effect strength for different *N* (as indicated on the *x*-axis). Each panel presents results for a different method of differential expression calling, while different colours correspond to different methods for expression estimation (see legend). With the additional filters, there is generally good agreement across sites for the 1000 top ranked genes, for all methods. The reliability with which methods identify short lists of the 50–100 genes of highest interest with the strongest effect size (largest fold change), however, varies considerably. Such variation in performance can be understood as resulting from the different assumptions and models underlying each computational analysis pipeline, including both the steps of estimating expression levels and of finally making differential expression calls (involving explicit or implicit noise models, *ℓ*).

## Discussion and conclusions

High-throughput expression profiling is a fast moving field both in terms of innovation in measurement technology as well as advances on the data analysis side. Especially for RNA-seq a plethora of new tools is being developed, and the selection of an effective pipeline is not trivial [24]. Going beyond the comparisons of the original SEQC study [2, 3], we here present comprehensive benchmark results covering all known genes and a range of effect sizes typically observed in experiments. The different expression level distributions observed in experiments reflect systemic traits of biological samples and any influence of hidden factors connected with site or protocol related variations. Differences in the characteristics of signal noise and bias may then affect the performance of specific methods for differential expression analysis, depending on their underlying statistical models. We hence report in detail on the observed sensitivity, specificity, and reproducibility of a range of popular computational methods for differential expression analysis by RNA-seq.

The sensitivity was in general determined by the chosen approach for expression level estimation, with the corresponding effect dominating over any variation due to method choice for differential expression calls (two-way ANOVA, *p*<5*%*). An analysis of results in the context of related experiments allowed the application of modern tools [16, 17] to identify and remove hidden confounders, yielding a much improved eFDR without affecting the overall sensitivity landscape. Thus, we have demonstrated the effectiveness of factor analysis for compensating site-specific artefacts. Reliable differential expression calls from RNA-seq, however, still required additional filters of genes with low abundances or small effect strengths, in order to address initially high rates of false positives. We could demonstrate clear and drastic improvements for both genome-scale surveys as well as the identification of genes with strong expression changes, giving prioritized candidates for further investigation. Notably, with the appropriate filters we could achieve good agreement across sites and also between different pipelines, making algorithm choice less critical in general.

Still, our benchmark results bear out a number of trends, and comparisons thus support several conservative recommendations. Notwithstanding the potential utility for transcript discovery, pipelines relying on TopHat2/Cufflinks2 for an estimation of expression levels performed the worst, while newer tools such as BitSeq or kallisto in general performed better. It is noteworthy that even when novel transcript discovery is desired, better performance can be obtained by a separate discovery step and subsequent quantification and differential expression analysis for known and newly identified transcripts [8]. For identification of the top-ranked differentially expressed genes, DESeq2 reliably performed well. Prioritization of candidates for further examination typically focuses on the most strongly differentially expressed genes. For the top-ranked genes, a combination of kallisto or BitSeq with DESeq2, factor analysis, and additional filters performed particularly well.

## Outlook

In a comparative benchmark extending the FDA SEQC reference study we identified effective RNA-seq data processing pipelines with the best performance in differential expression profiling. We could achieve a substantial improvement of specificity and reproducibility – all the while maintaining good sensitivity. While this report focused on differential expression at the gene level, RNA-seq also allows the analysis of alternative gene transcripts. Although the functional relevance of alternative transcripts has long been recognized [25], a large fraction are only weakly expressed. This brings additional challenges in dealing with the disambiguation of reads, sequencing noise, and biases in the estimation of expression levels and differential analysis. Consequently, a study of the sensitivity, specificity, and reproducibility of differential expression profiling that discriminates alternative transcripts is beyond the scope of this study and will be examined elsewhere.

## Reviewers’ comments

### Reviewer’s report 1: Michał Okoniewski, PhD

### ID Scientific IT Services, ETH Zürich

The manuscript by P. Łabaj and D. Kreil is a creative and educative extension of the SEQC study. The SEQC was designed to be a multi-lab effort and to prove the utility of RNA-seq, which was finally successful and presented the many-sided view of the data analysis, interpretation and use in biomedical research.

The study was so rich in information, that the main architects of it as well as external researchers can still find additional gems of knowledge doing a creative re-analysis of the datasets. In this case, the authors undertook the non-trivial challenge of running in a systematic way several major types of analysis and comparing them in terms of differentially expressed genes using intersection and unions of the lists of genes.

Conceptually, it is non-trivial to design the analysis in such a way that it is possible, because the analysis pipelines have various approach to primary (alignment) and secondary (statistical) analysis as well as the output of the tools is normally not directly comparable. Still, the authors solved those data science challenges successfully and could follow up with additional data analysis experiments for to compare the methods and use of additional tools improving the outcome, such as factor analysis or making the final gene lists more precise by filtering.

The authors did also good job in selecting the appropriate dataset, which included more than usual amount of “subtle” gene expression changes, that do not have a high fold change, but should be detectable by clever statistical methods.

The main advantages of the manuscript are: the overview and comparison across methods and the educative results and good practices on making the RNA-seq more precise - as one of the main problems here is the lack of objective reference together with the need to find the “biological truth”.

Authors are definitely experts in the area, so most deficiencies of the manuscript come form the fact that certain aspects are too obvious for them and are explained too briefly or not at all. This can be at times problematic for the educational aspect of the paper, but not for the findings and all those are in fact minor issues. In particular:

- (abstract) would be worth mentioning at once that this is data analysis benchmark (not eg RNA-seq wet-lab one)
  Author response: *We have extended the text accordingly:* “***In our benchmark of alternative tools for RNA-seq data analysis*** *we demonstrate the benefits that can be gained, in particular, by analysing results in the context of other experiments employing a reference standard sample.”*
- (abstract) abbreviation “eFDR” is introduced without explanation. It happens also to some other abbreviations, eg “POG” in Fig 6.
  Author response: *We have extended the text accordingly: “In itself, this already substantially improves the* ***empirical False Discovery Rate (eFDR)*** *without changing the overall landscape of sensitivity.” and “The y-axis plots the* ***percentage of genes (POG)*** *identified as differentially expressed in the same direction and with significance in both alternative sites compared.”*
- (abstract) would be good to tell somehow more descriptively what is reproducibility in this context
  Author response: “*With artefacts removed by factor analysis and additional filters, for genome scale surveys, the reproducibility* ***of differential expression calls*** *typically exceeds 80% for all tool combinations examined.”*
- (abstract) “analysing results in the context of other experiments” - is not clear and slightly misleading
  Author response: *We have modified the text accordingly: “In our benchmark of alternative tools for RNA-seq data analysis we demonstrate the benefits that can be gained, n particular,* ***by analysing results in the context of other experiments employing a reference standard sample*** *.”*
- (introduction) would be good to explain more why “considering subtle signals” is important
  Author response: *We have extended the text accordingly: “This pair of samples has the smallest average effect strength (‘signal’) amongst the different possible pair-wise comparisons of the MAQC/SEQC A, B, C, and D, allowing us to* ***also consider performance for more subtle signals, such as expression changes for typically weakly expressed molecular switches*** *.”*
- (introduction) would be good to explain more why AceView was chosen for gene models, not eg. Ensembl or NCBI RefSeq
  Author response: *We have added the according explanation in the* Methods *section: “* ***In this study the AceView gene models have been used. We previously have shown that, despite its age, AceView remains the most comprehensive and accurate annotation database for human*** *.”*
- (introduction and methods) the authors should not assume the knowledge of details of SEQC study and explain what samples A and C are
  Author response: *We have added the extended explanation in the* Methods *section: “This study builds on the main synthetic benchmark data set of the SEQC consortium, where known mixtures of standardized reference samples have been sequenced by multiple platforms in a setup controlling for laboratory site specific effects.* ***In particular, the well-characterized reference RNA samples A (Universal Human Reference RNA) and B (Human Brain Reference RNA) from the MAQC consortium have been used. Samples A and B were then mixed in known ratios, 3:1 and 1:3, to construct samples C and D, respectively. In this data analysis benchmark*** *our results are based on the subset of samples A and C at six Illumina HiSeq 2000 sites* ***where each sample has been sequences with 4 technical replicates***.*”*
- (methods) what were the parameters of counting in r-make and subread?
  Author response: *Details of how the tools have been run can be found in the supplement to the SEQC manuscript (doi:10.1038/nbt.2957), and the revised text states: “Details of how all tools were run can be found in the Supplementary materials of the original SEQC/MAQC-III study; kallisto has been used with default parameters.”*
- (methods) the explanation on what tophat -G does is not very clear and accurate
  Author response: *We have extended the explanation of what TopHat does when used with -G option: “The popular* TopHat2 *tool with the ‘–G’ option pursues a hybrid approach,* ***where based on the provided gene models the virtual transcriptome is constructed and reads are first aligned to it, in line with our analyses showing that this improves the precision of expression estimates. In the next steps these aligned reads are mapped back to the genome and the remaining not aligned yet reads are aligned to the genome sequences*** *.”*
- (methods) BitSeq as “provided by SHRiMP2” is also not appropriate description
  Author response: *We have improved the method description accordingly: “In contrast,* BitSeq *directly uses the* ***transcriptome alignments (here we have aligned the reads to the transcriptome with use of*** SHRiMP2 ***)*** *to assess transcript abundances.”*
- (methods) Perhaps more precise and explicit categorisation of the tools would be informative. A schema/data-flow of the workflows with data formats, tools and output integration/comparison methods would help to understand this section
  Author response: *The Supplementary Fig. S1 provides the requested schema.*
- (methods) citing sailfish would be useful, even if kallisto was chosen as representative in this software category
  Author response: *Appropriate reference has been added.*
- (results) the first paragraph is in fact repeated story of methods
  Author response: *As accurately observed by the reviewer, the first paragraph of the Results section intends to summarize the scope of the reported benchmark work. A detailed description of the tools is then provided in the* Methods *section.*
- (results) perhaps an explanation of the need for small expression level filters would be informative
  Author response: *We have extended the section in question accordingly: “For RNA-seq, unlike for microarrays, beyond filters for small effect size (fold change) also filters for small expression levels are necessary.* ***This is needed in order to remove False Positives arising from the large scatter weakly expressed transcripts, which can be seen as a ’comet head’ in typical M(A) plots (cf. Fig. 2)*** *.”*
- (results - effects on implicated genes) would be good if authors could at least hypothesise what the sources of disagreement in the methods come from, as this is a typical conceptual problem for RNA-seq analysis beginners
  Author response: *We have added the possible explanation at the end of the subsection: “* ***Such variation in performance can be understood as resulting from the different assumptions and models underlying each computational analysis pipeline, including both the steps of estimating expression levels and of finally making differential expression calls (involving explicit or implicit noise models, ℓ)*** *.”*
- (conclusions) criticising tophat/cufflinks is probably too harsh, as the primary purpose of cufflinks is novel transcript discovery, not the quantitation
  Author response: *We agree with the reviewer, in that early versions the Cufflinks may have been developed with the primary aim of novel transcript discovery. The software then has developed into a very popular tool not only for transcript discovery but also for expression quantification and differential expression calling. On the Cufflinks webpage, the first sentence already states: “Cufflinks assembles transcripts, estimates their abundances, and tests for differential expression and regulation in RNA-seq samples.” We have revised our text to clarify: “* ***Notwithstanding the potential utility for transcript discovery,*** *pipelines relying on* TopHat2/Cufflinks2 *for an estimation of expression levels performed the worst, while newer tools such as* BitSeq *or* kallisto *performed better.”*

### Reviewer’s report 2: Charlotte Soneson, PhD

### Institute of Molecular Life Sciences, University of Zurich

In this manuscript, Labaj and Kreil are comparing various abundance estimation and differential expression pipelines using RNA-seq data from the SEQC consortium. They consider five abundance estimation methods and three differential expression approaches, covering a large part of the most common workflows used in practice. The main conclusions are that the sensitivity is mainly dependent on the choice of abundance estimation method, and that accounting for hidden confounders together with filtering out genes with low abundance or fold change can improve FDR control and agreement across methods and experiments.

The manuscript has the potential of being informative to the community, but would benefit from a better description of the data as well as the employed methodology. For example:

- a more thorough description of the subset of the SEQC data that was used (number of replicates from each site, type of replicate (technical)).
  Author response: *We have added the extended explanation in the* Methods *section: “This study builds on the main synthetic benchmark data set of the SEQC consortium, where known mixtures of standardized reference samples have been sequenced by multiple platforms in a setup controlling for laboratory site specific effects.* ***In particular, the well-characterized reference RNA samples A (Universal Human Reference RNA) and B (Human Brain Reference RNA) from the MAQC consortium have been used. Samples A and B were then mixed in known ratios, 3:1 and 1:3, to construct samples C and D, respectively. In this data analysis benchmark*** *our results are based on the subset of samples A and C at six Illumina HiSeq 2000 sites* ***where each sample has been sequenced with 4 technical replicates*** *.”*
- it would be very useful to have (e.g.) an R markdown file outlining the whole analysis. That would, for example, make it unambiguous what is meant by “default settings” for the differential expression calling methods and precisely how svaseq was applied.
  Author response: *The appropriate R code has been provided as Supplementary Material in Additional file* 1.
- how were abundances from kallisto/BitSeq combined into gene-level “read count equivalents”? I.e., were the estimated read counts summarized directly, or were TPM estimates aggregated and then scaled to read count equivalents? This could potentially make a big difference, especially in the presence of differential transcript usage between conditions.
  Author response: *For pipelines where only transcript expression abundances are provided (BitSeq and kallisto), gene-level ‘read count equivalents’ were obtained by summing up the transcript-level ‘read counts equivalents’. Considering that different approaches can result not only in differences in expression estimates but also in varying differential expression calls, we treated all tools alike to obtain (estimated) read counts without any conversions. We are well aware of the fundamental differences between ‘raw reads counts’, R/FPKM, RPM and TPM measures, and these were extensively studied already elsewhere [e.g. Dillies et al. (2012) Brief in Bioinf 14(6):671-683; Wagner at al. (2012) Theory in Bioscience, 131:281]. In this manuscript we use ‘raw read counts’ or ‘raw read equivalents’ (for tools providing expression estimates for alternative transcripts not genes) on one hand for simplicity, and on the other hand to facilitate a fair comparison of the alternative differential expression methods. Many of these were originally designed for ‘raw read counts’ by their authors.*
- it could be clearer that the focus is on genes that are downregulated in the A sample (it says just “downregulated”).
  Author response: *Text has been adjusted accordingly. “In differential expression analysis of samples A/C we can focus* ***on genes down-regulated in sample A*** *because the effect strength of any potential up-regulation is limited to* ***a maximum of*** *a 4/3-fold increase by design, as sample C is 3 parts of sample A and one part of sample B. We therefore expect no up-regulated genes satisfying commonly used thresholds for effect strength.”*
- what does it mean that “the direction of fold change is taken into account” for the calculation of inter-site reproducibility? If a gene is considered upregulated at one site and downregulated at another, is it counted twice in the union of the lists?
  Author response: *In case of situation when gene is considered upregulated at one site and downregulated at another, this gene is not counted as agreed between the sites, although being on both lists of (topN) differentially calling genes. We have modified the description to make this point clearer. “The overall agreement between lists of differentially expressed genes has been calculated as the ratio of list intersection and list union. The agreement of the top N candidates has been calculated as the ratio of the length of the intersection of the top N genes from the compared lists (differentially expressed candidates have been order by effect strength) divided by N.* ***The direction of fold change is taken into account: genes showing opposite directions of change are considered not to agree, and are thus excluded for computing the list intersection assessing agreement. All gene lists are sets, either including or excluding gene names, with no gene counted more than once*** *.”*
- how were the M- and A-values shown in Fig. 2 determined? Are they taken from the output of one of the differential expression methods or calculated independently of these?
  Author response: *For a comparative visualization of differential expression calls across methods, on a canvas of M- and A- values from limma, we contrast which genes have specifically been called as a differentially expressed by individual methods. While the choice of M(A) values from limma is in a sense arbitrary, as M- and A- values of specific genes differ between methods because of different internal normalization procedures, some common values needed to be chosen for purposes of comparative display, with no effect on qualitative results.*
- how, precisely, was the eFDR calculated?
  Author response: *We have added a dedicated subsection in the* Methods: “***Taking advantage of the SEQC study design we can infer an empirical False Discovery Rate (eFDR) by comparing the amount of genes identified as ‘differentially expressed’ in the cross-site same–same comparison (A-*** ***vs*** ***-A and C-*** ***vs*** ***-C) with differentially expressed genes in the A-*** ***vs*** ***-C comparison: eFDR=(A***_***1***_ ***−vs−A***_***2***_ ***+C***_***1***_ ***−vs−C***_***2***_ ***)/(A***_***1***_ ***−vs−C***_***2***_ ***+A***_***2***_ ***−vs−C***_***1***_ ***), where: X***_***N***_ ***−vs−Y***_***M***_ ***is the number of genes identified as differentially expressed when comparing sample*** ***X*** ***from site*** ***N*** ***with sample*** ***Y*** ***from site*** ***M***.”
- which values were used to perform the abundance filtering? The average (normalized?) counts across all samples?
  Author response: *The M and A values computed in each specific pipeline have been used for filtering.*
- DESeq2 performs a filtering of lowly abundant genes by default. How does that automatically determined threshold compare to the threshold imposed by the explicit abundance filter applied by the authors?
  Author response: *The reviewer has raised a very interesting question. The focus of this manuscript, however, was in a comparison of tools employed with their default settings as recommended by their authors. We will further investigate this idea in future work.*
- Minor points:
  - In the last sentence of the Discussion, “will ve” should be “will be”
  - In table 2, should the last sentence read “absolute log-fold change larger than one” rather than “absolute log-fold change larger than two”?
  - In figures 3 and 5, the y-axis label says [tousand] instead of [thousand]
  Author response: *The pointed out typos have been corrected. We also have double checked the rest of the text to eliminate other mistakes and typos.*

## Additional file

Additional file 1 Additional file 1 shows a schema of how the examined tools were employed. Moreover, the R code is provided that was used to run svaseq and call the various tools for differential expression analysis. (PDF 262 kb)

## Abbreviations

AE: Average expreesion level
DEC: Differential expression calling tool/method
DEG: Differentially expressed genes
EE: Expression estimate tool/method
eFDR: Empirical False Discovery rate
FC: Fold change
MAQC-III: third phase of Microarray Quality Control project
POG: Percent of overlapping genes
SEQC: Sequencing Quality Control project
