# Supplementary material for: Sensitivity, specificity, and reproducibility of RNA-Seq differential expression calls
Source: Biol Direct. 2016 Dec 20;11:66. doi: 10.1186/s13062-016-0169-7 (PMC5168849; doi:10.1186/s13062-016-0169-7)
Supplement: Additional file 1 — shows a schema of how the examined tools were employed. Moreover, the R code is provided that was used to run svaseq and call the various tools for differential expression analysis. (PDF 262 kb) [file 13062_2016_169_MOESM1_ESM.pdf]

Supplementary Figure

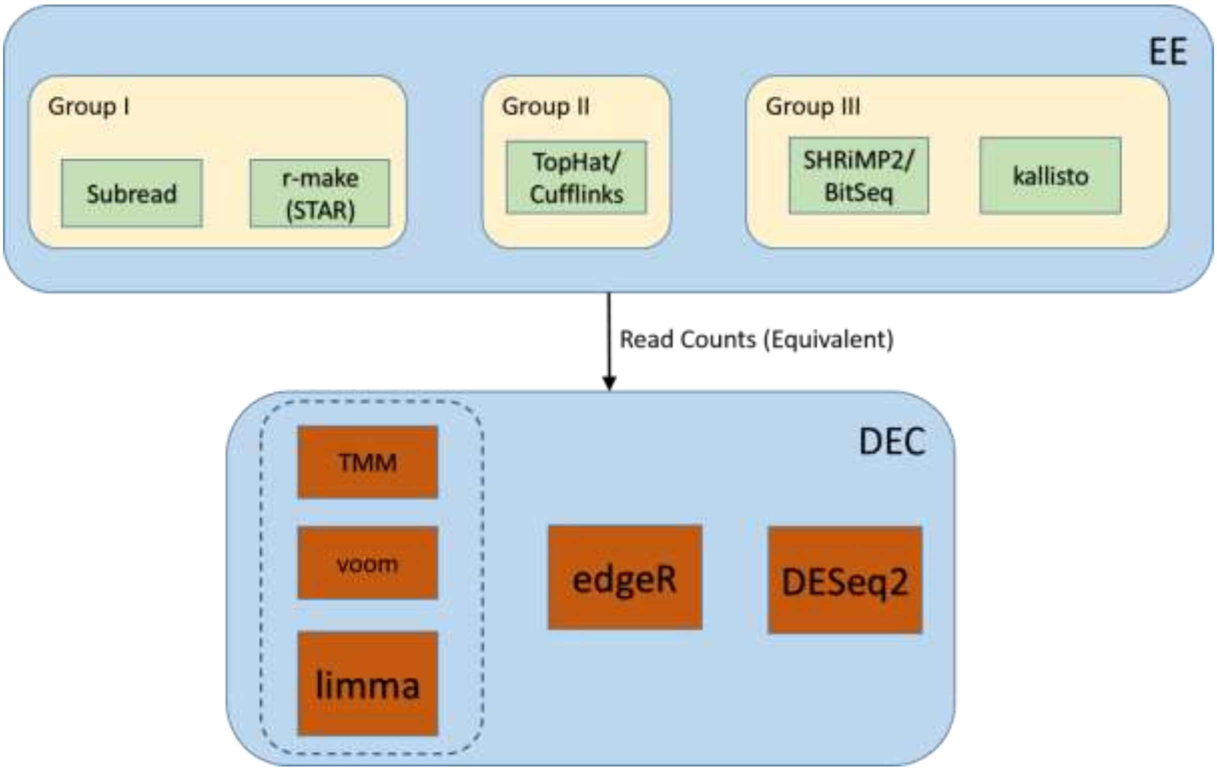

Figure S1. Schema presenting the groups represented by the examined tools.

The examined tools estimating the expression (EE) can be seen as representatives of three groups.

I) Tools which perform and alignment of the sequenced reads to the genome, followed by counting reads that fall into the known gene regions.

II) Tools from hybrid approach, where based on the provided gene model the virtual transcriptome is constructed and reads are first aligned to it. In the next steps these aligned reads are mapped back to the genome and the remaining not aligned yet reads are aligned to the genome sequences (TopHat). Gene and transcript expression levels are then estimated using the tool that processes the genome-based alignments (Cufflinks).

III) Tools which perform (pseudo) alignment of the sequenced reads to the alternative transcript sequences and then asses the alternative transcripts abundances. The gene level abundances are obtained by summing the ones from the alternative transcripts.

Read counts or read count equivalents provided by the EE tools are then fed to differential expression calling (DEC) tools. Here three popular alternatives have been examined: limma (together with TMM and voom), edgeR, and DESeq2.

Supplementary R code

Factor analysis by SVA

```
samples.fc <- c()
for(x in colnames(data.in))
  {
    sampl <- substr(x, 5,5)
    samples.fc <- c(samples.fc, sampl)
  }
group = as.factor(samples.fc)
mod1 = model.matrix(~group)
res <- svaseq(data.in, mod=mod1,  constant=0.5, B=5, n.sv=4)
new.dat <- fsva(log(data.in+0.5), mod1, res, newdat=data.in, method="exact")
data.out <-exp(new.dat$db)-0.5
```

Differential expression analysis

```
DEfun <- function(counts, design)
  {
    DE <- list()


## limma
    gene.dge <-  DGEList(counts=counts, group=factor(rep(1:2, each=4)))
    gene.dge.norm <- calcNormFactors(gene.dge)

    gene.dge.norm2 <- gene.dge.norm
    gene.dge.norm2$counts <- gene.dge.norm2$counts-0.5
    rpm <- voom(gene.dge.norm2, design)


    cm <- makeContrasts(AvsC=As-Cs,
                    levels=design)
    fit <- lmFit(rpm,design)
    cf <- contrasts.fit(fit, cm)
    fit.eB <- eBayes(cf, proportion=0.3, robust=TRUE)
    adjust.meth <- "BH"
    tt.limma <- topTable(fit.eB, adjust.method=adjust.meth,number=Inf)
    FC <-tt.limma$logFC
    AE <-tt.limma$AveExpr
    qval <- tt.limma$adj.P.Val
    names(FC) <- names(AE) <- names(qval) <- rownames(tt.limma)
    DE.list <- DElist(FC, AE, qval, 0.05)
    DE[["limma"]] <- list(DE.list=DE.list)

## edgeR
    y <- estimateCommonDisp(gene.dge.norm)
    y <- estimateTagwiseDisp(y)
    et <- exactTest(y)
    tt.edgeR <- topTags(et, n=Inf, adjust.method=adjust.meth)
    FC <--tt.edgeR$table$logFC
    AE <-tt.edgeR$table$logCPM
    qval <- tt.edgeR$table$FDR
    names(FC) <- names(AE) <- names(qval) <- rownames(tt.edgeR$table)
    DE.list <- DElist(FC, AE, qval, 0.05)
    ## DE[["edgeR"]] <- list(fit=et, tt=tt.edgeR$table, DE.list=DE.list)
    DE[["edgeR"]] <- list(DE.list=DE.list)

## DESeq2
    colData <- data.frame(condition=rep(c("A","C"), each=4), type="paired-end")
    rownames(colData) <- colnames(counts)

    dds <- DESeqDataSetFromMatrix(countData = round(counts),
                              colData = colData,
                              design = ~condition)
```

```
    dds <- DESeq(dds)
    tt.DESeq2      <-      DESeq2::results(dds,      contrast=c("condition","A","C"),
pAdjustMethod = "BH",)
    FC <-tt.DESeq2$log2FoldChange
    FC[is.na(FC)] <- 0
    AE <-log2(tt.DESeq2$baseMean+0.5)
    qval <- tt.DESeq2$padj
    qval[is.na(qval)] <- 1
    names(FC) <- names(AE) <- names(qval) <- rownames(tt.DESeq2)
    DE.list <- DElist(FC, AE, qval, 0.05)
    DE[["DESeq2"]] <- list(DE.list=DE.list)

    return(DE)
  }
```
